# The untapped potential of actinobacterial lanthipeptides as therapeutic agents

**DOI:** 10.1007/s11033-023-08880-w

**Published:** 2023-11-07

**Authors:** Dulce Ramírez-Rendón, Fernando Guzmán-Chávez, Carlos García-Ausencio, Romina Rodríguez-Sanoja, Sergio Sánchez

**Affiliations:** Instituto de Investigaciones Biomédicas, Universidad Nacional Autónoma de México, CDMX, 04510 Mexico City, México

**Keywords:** Lanthipeptides, *Actinobacteria*, Natural products, Antibiotic-resistant

## Abstract

The increase in bacterial resistance generated by the indiscriminate use of antibiotics in medical practice set new challenges for discovering bioactive natural products as alternatives for therapeutics. Lanthipeptides are an attractive natural product group that has been only partially explored and shows engaging biological activities. These molecules are small peptides with potential application as therapeutic agents. Some members show antibiotic activity against problematic drug-resistant pathogens and against a wide variety of viruses. Nevertheless, their biological activities are not restricted to antimicrobials, as their contribution to the treatment of cystic fibrosis, cancer, pain symptoms, control of inflammation, and blood pressure has been demonstrated. The study of biosynthetic gene clusters through genome mining has contributed to accelerating the discovery, enlargement, and diversification of this group of natural products. In this review, we provide insight into the recent advances in the development and research of actinobacterial lanthipeptides that hold great potential as therapeutics.

## Introduction

The discovery of novel antimicrobial compounds with potential application as novel drugs is necessary to face the increase of bacterial resistance to antibiotics currently used to treat bacterial infections in clinical facilities. This situation poses a serious global concern and a major threat to global public health. Thus, the increasing emergence of resistant strains demands the design of novel therapeutic strategies [1]. Among the different families of natural products, the ribosomally synthesized and post-translationally modified peptides (RiPPs) are important antimicrobial compounds with diverse potential activities and applications in the clinic and food industry. RiPPs present a wide structural diversity and comprise small peptides produced from a precursor peptide that is later extensively post-translationally modified (PTMs) by biosynthetic enzymes to yield the mature bioactive product [2]. Two regions primarily integrate the precursor peptide, an N-terminal leader peptide, that can act as a secretion signal and contains a sequence necessary for recognition by biosynthetic enzymes. The other region is a C-terminal core peptide where post-translational modifications are installed [3]. The discovery and study of novel RiPPs have been impacted by the advances in DNA sequencing technologies and the publication of the genome sequences of potential secondary metabolites producer bacteria. Likewise, the constant improvement of databases and automated bioinformatic tools for predicting and annotating biosynthetic gene clusters (BGCs) have also influenced this discovery disclosing large chemical diversity [4, 5]. In this sense, genomic mining has become a key tool for discovering the biosynthetic machinery for novel RiPPs [6].

The *Actinobacteria* phylum is a prolific source of biologically active compounds. Their biosynthetic potential stands out from their ability to produce antibiotics, antifungals, anticancer, and biocontrol agents [7–10] and has historically contributed to human health. More than 45% of the currently described microbial bioactive compounds are of actinobacterial origin, and their products show an enormous chemical diversity [5]. Lanthipeptides from *Actinobacteria* present unique characteristics often related to a higher biological activity. Among them, halogenation is present in some compounds like microbisporicin [11], or hydroxylations in lanthipeptides of the cinnamycin group and cebulantin [12]. Likewise, aminovinylcysteine (AvyCys) has been widely described in actinobacterial lanthipeptides like epidermin [13]. To date, just a limited range of molecules, such as bottromycins, linear azole-containing peptides, thiopeptides, lasso peptides, linaridins, and lanthipeptides, have been described as molecules with potential biological activity in these microorganisms. These compounds, usually with broad antimicrobial activity against Gram-positive bacteria, also include those resistant to 𝛽-lactams and glycopeptides [3, 6, 14]. Besides, there are actinomycetes showing considerable RiPPs cryptic clusters in their genomes that need to be unveiled to evaluate their potential for therapeutical applications. Among those molecules, the lanthipeptides are the most extensively compounds studied [15].

**Fig. 1:**
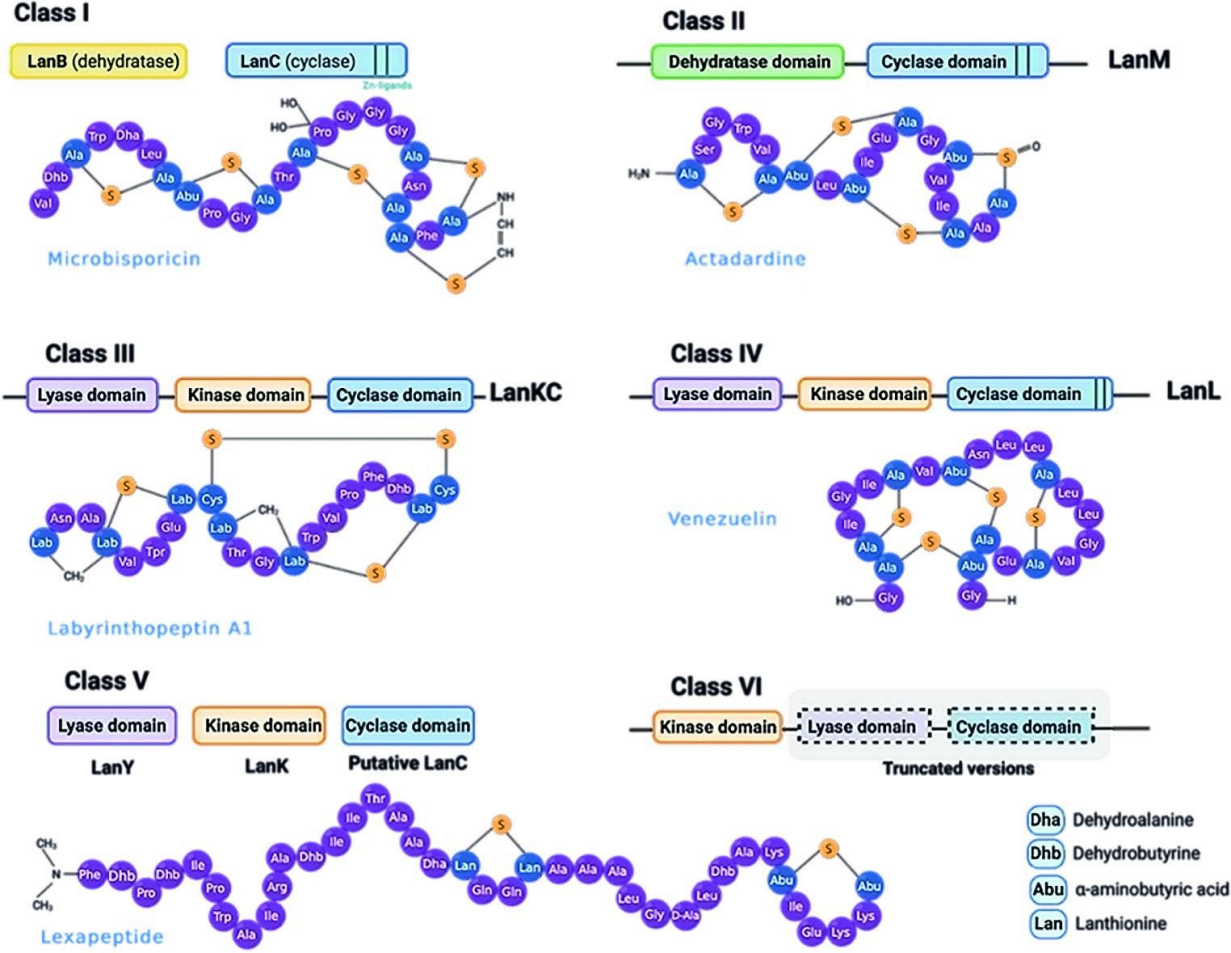
Classification of lanthipeptide synthetases and representative structures of the post-translational modifications in lanthipeptides produced by *Actinobacteria*. Class VI lanthipeptide synthetases have been only identified in *Streptococcus* spp.

Since the discovery of nisin in 1928, dozens of lanthipeptides with a broad diversity of structures have been reported harboring thioether cross-linked amino acids (MeLan: 3-methyl-lanthionine or Lan: Lanthionine) [6, 16]. Despite this structural feature, the lanthipeptides have been categorized into five groups based on the biosynthetic machinery displayed for building the (Me)Lan units (Fig. 1). In class I lanthipeptides, the lanthionine cross-link form is generated by the concerted action of two lanthionine synthetases, LanB and LanC, which catalyze the dehydration and cyclization reactions, respectively [6, 17]. Whereas, in class II, III, and IV lanthipeptides, these two reactions are carried out by a multifunctional lanthipeptide synthetase (LanM, LanKC, and LanL, respectively) [16, 18], whose main difference is the absence of the conserved zinc-binding motif (Cys-Cys-His/Cys) in class III enzymes (LanKC). Interestingly, this motif is also present in the cyclase LanC from class I, where the zinc allows the nucleophilic attack on the dehydroamino acids for activating the Cys thiols [19, 20]. In addition, two new lanthipeptide synthase classes (V and VI) were discovered in recent years [16, 21]. The class V lanthipeptides contain three independent enzymes with lyase (LanY), kinase (LanK), and cyclase (putative LanC) activity [21, 22]. In contrast, class VI harbors a lanthionine synthetase with a kinase and the truncated cyclase and lyase domains. Notably, this class has only been reported in *Streptococcus* spp. [16]. By conducting in vitro studies, these authors found that in class VI lanthipeptides, the substrate is short, and a leader peptide guides the process to produce miniature lanthipeptides with a 4 amino acids ring. However, further studies on these miniature RiPPs are required to unveil their precise function (Fig. 1).

In general, the mechanism of action of lanthipeptides that show antimicrobial activity (lantibiotics) is based on binding to lipid II (undecaprenyl-pyrophosphoryl-MurNAc-(pentapeptide)-GlcNAc), a highly conserved peptidoglycan precursor in the cytoplasmic membrane of bacteria [23]. Additionally, this interaction with lipid II leads to pores formation in the cell membrane, provoking the release of cellular content in Gram-positive bacteria [15, 24].

**Fig. 2:**
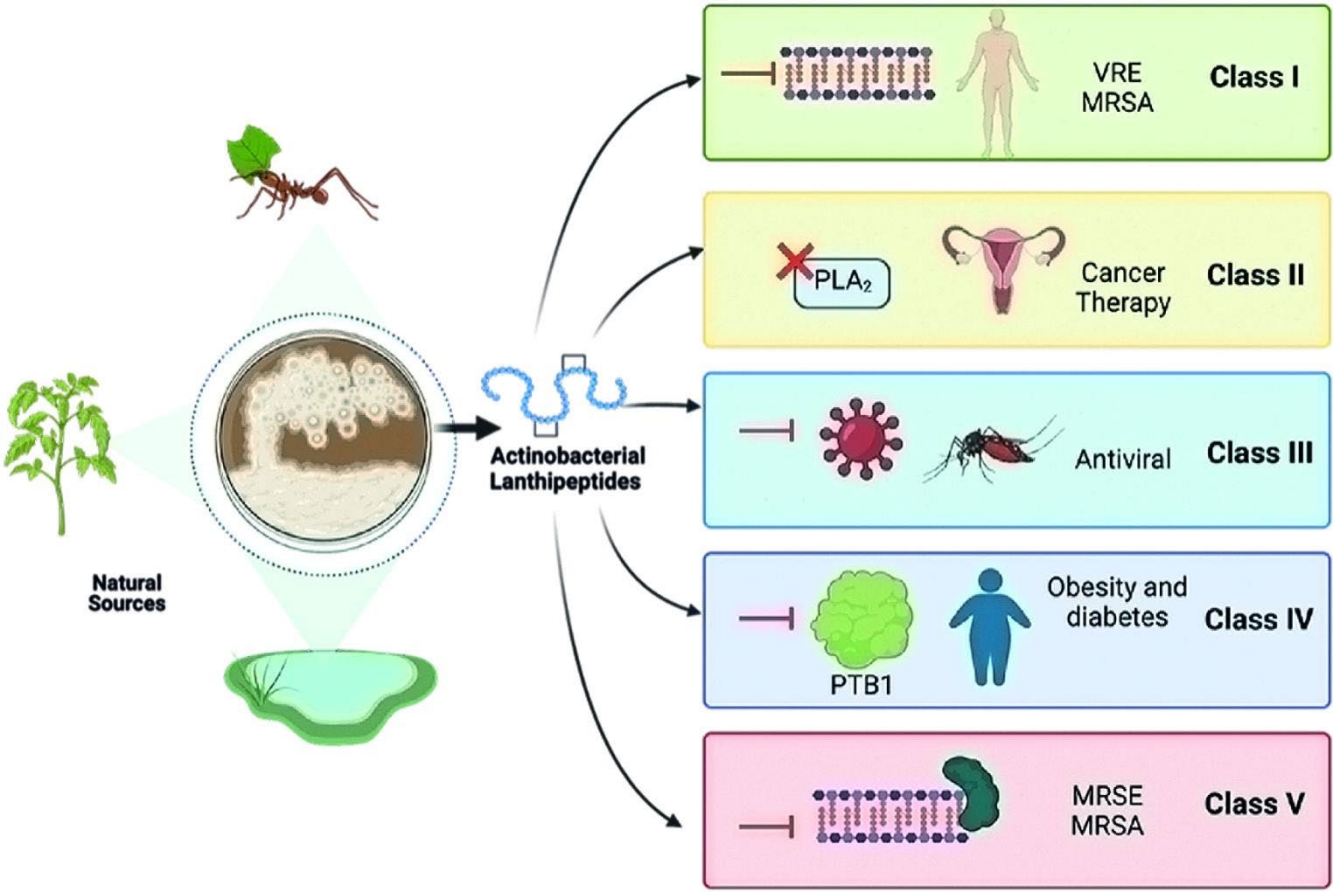
Actinobacterial lanthipeptides classes. *Actinobacteria* producers of active lanthipeptides are widely distributed in different ecosystems, such as plants, insects, and water bodies. Based on the biosynthetic machinery used for building the (Me)Lan units, the lanthipeptides have been classified into five groups, where a wide range of potential therapeutic targets have been observed. VRE: vancomycin-resistant Enterococci. MRSA: Methicillin-resistant *Staphylococcus aureus.*

Here, we summarize the most representative Actinobacterial lanthipeptides of each class with special attention on their application as potential therapeutics (Fig. 2). Likewise, a brief description of new strategies to discover novel lanthipeptides is provided.

### Class I lanthipeptides

#### Microbisporicin

Microbisporicin, also known as NAI-107, is a 24 amino acid lantibiotic identified during the screening program designed to find bacterial cell wall inhibitors [25]. It is produced by *Microbispora* sp. 107,891, *Microbispora corallina* NRRL 30,420 [25] and *Actinoallomurus* spp. [26]. Microbisporicin contains one methyl-lanthionine, three lanthionines, and a C-terminal aminovinylcysteine (AviCys). Additionally, it includes the unusually modified amino acids 3,4-dihydroxyproline and 5-chlorotryptophan, which had not been detected before in lantibiotics. It is produced as a complex of related congeners molecules that varies between them by the presence of zero, one or two hydroxyl groups at Pro-14, a chlorine at Trp-4, and a sulfoxide on the thioether of the first lanthionine [27]. The most studied microbisporicin congeners A1 and A2, differ between them in the amino acid in position 14; 3,4-dihydroxy-proline or 4-hydroxy-proline, and have a molecular weight of 2246 and 2230 Da, respectively [25]. Adding KBr to the medium generated the brominated variant NAI-108 with slightly improved antibacterial activity [28]. This lantibiotic inhibits cell wall biosynthesis and impacts membrane functionality by binding to bactoprenol-pyrophosphate-coupled precursors of the bacterial cell wall and thus forming 1:1 and 2:1 complex (peptide: lipid II), the N-terminal region is presumably responsible for the interaction with the pyrophosphate moiety of lipid II [29], displaying antimicrobial activity against a wide range of Gram-positive pathogens including multi-drug resistant bacteria of medical importance such as methicillin-resistant *Staphylococcus aureus* (MRSA), glycopeptide-intermediate resistant *S. aureus* (GISA), vancomycin-resistant Enterococci (VRE), penicillin-resistant *Streptococcus pneumoniae* [29], some *Clostridia* and *Propionibacterium*. Regarding Gram-negative bacteria, microbisporicin shows antibacterial activity against *Neisseria meningitidis*, *Moraxella catarrhalis*, and *Haemophilus influenzae* [30]. This activity likely results from an increased net charge from halogenation in the lanthipeptide structure, increasing cellular penetrability. However, additional proposals involve changes in the outer membrane cellular permeability. Thus, more studies are needed to clarify this situation [29, 31]. The activity of microbisporicin is comparable to or better than nisin, the most extensively studied lantibiotic, and the reference antibiotics like vancomycin and teicoplanin. Furthermore, microbisporicin and NAI-108 are highly active against *Neisseria gonorrhoeae*, including penicillin-resistant clinical isolates.

It is noteworthy to mention the strong synergistic effect that microbisporicin shows in combination with the antibiotic polymyxin against Gram-negative pathogens [25, 30] and the excellent efficacy in several murine infection models induced by drug-resistant Gram-positive pathogens with efficacy comparable or superior to reference compounds. The rapid bactericidal activity and its prolonged half-life in plasma is an important trait of this lantibiotic, making it a valuable antibiotic candidate to tackle the problem of antimicrobial resistance in Gram-positive bacteria.

NAI-107 is currently in the late preclinical stage [32]. However, further studies for applying microbisporicin in treating infections caused by multi-resistant pathogens must be carried out [25, 30, 32–34].

#### Planosporicin

Planosporicin is a lantibiotic produced by the actinomycetes *Planomonospora alba*, and *Planomonospora* sp. strain DSM 14,920, and is encoded by a large biosynthetic cluster of 15 genes [35]. This lanthipeptide is a 2194 Da polypeptide with a globular structure that harbors four lanthionine and one methyl-lanthionine bridge [27]. Its mechanism of action is like other class I lanthipeptides, providing activity against multi-resistant Gram-positive pathogens like *Staphylococcus aureus*, *Streptococcus pyogenes*, *Streptococcus pneumoniae*, *Lactobacillus garviae*, *Clostridium perfringens, Clostridium difficile, and Moraxella catarrhalis* [11, 36].

#### Cebulantin

Cebulantin is a lanthipeptide described by an analysis known as HiTES (high-throughput elicitor screening) [37], where the drugs furosemide and fenofibrate were used as elicitor agents to induce cebulantin production in the actinomycete *Saccharopolyspora cebuensis*. This lantibiotic of 22-amino acid residues is made up of one lanthionine and two methylthionine rings with a hydroxy group at Pro-13. The most outstanding aspect of this lantibiotic is its activity against Gram-negative pathogens, such as cell-wall-weakened *Escherichia coli* (ΔlptD), as well as against several *Vibrio* strains, like *V. parahaemolyticus* [38]. There are no scientific arguments to explain the affinity of cebulantin by Gram-negative bacteria. However, speculations include that it may be due to the presence of 4-OH-Pro in its structure [37].

### Class II lanthipeptides

#### Actagardine and its variants

Originally designated as gardimycin in 1976, actagardine is a 19-amino acid globular lantibiotic produced by *Actinoplanes garbadinensis* and *Actinoplanes liguriae* with a molecular weight of 1890 Da, which harbors an uncommon sulfoxide group and four intramolecular bridges in its structure, one lanthionine and three methyl-lanthionine [39] Additionally, some natural and semisynthetic variants of this lanthipeptide have been described, such as Ala(0)-actagardine which is a natural variant produced by *Actinoplanes liguriae* ATCC 31,048 that possess an additional N-terminal alanine [40]. The structural analog deoxyactagardin B (DAB) produced by *A. liguriae* NCIMB41362 differs from actagardin by the absence of the sulfoxide group and for the substitutions of two amino acids at residues 15 and 16, corresponding to Val and Ile in actagardin and Leu and Val in DAB [41]. These lanthipeptides generally display activity against Gram-positive pathogens like *Streptococcus* spp. and *Clostridium difficile* [42–44]. DAB shows a selective activity against *C. difficile*, thanks to the derivatization at its C-terminal end. It also exhibits reduced activity against normal intestinal microbiota, such as *Bacteroides* spp., *Prevotella* spp., *Porphyromonas* spp, *Lactobacillucasei*, and *L. rhamnosus*, showing higher MIC values than those reported against *C. difficile*. The selective effect against *C. difficile* has not been satisfactorily explained but seems to be related to the peptidoglycan necessary to maintain the cell wall integrity. Gram-negative bacteria have a thinner peptidoglycan layer than Gram-positive bacteria, which makes them more susceptible to the effects of actagardin and its variants [45, 46]. NVB-302 is another semisynthetic lantibiotic derived from DAB with a C-terminal 1, 7 diaminoheptane tail, whose modification improves the stability in simulated gastric fluid, bioactivity, and solubility of this compound. Regarding NVB-302, this lanthipeptide has completed phase I clinical trials for treating *C. difficile* and shows reduced activity against normal gut microbiota, disclosing the importance of generating lanthipeptide variants to improve certain characteristics [47–50].

NVB-333 is another example of a semisynthetic lantibiotic being tested in pre-clinical trials. This compound is produced by binding 3,5- dichlorobenzylamine at the C- terminal end of deoxyactagardine. Remarkably, it is not prone to resistance development and possesses activity against Gram-positive antibiotic-resistant pathogens such as *S. aureus* resistant to meticillin, vancomycin, linezolid and daptomycin, vancomycin-resistant *Enterococcus* and penicillin-resistant *S. pneumoniae*, which makes it a potential candidate in the treatment of infections caused by Gram-positive bacteria [34, 51, 52]. Similarly, NAI-802 is an actagardine analog of 21 amino acids isolated from *Actinoplanes* sp., which also displays activity against some Gram-positive anaerobic bacteria [41].

Additional potential applications for this lanthipeptide group are their use for plant disease control. For instance, michiganin A is a 21 amino acid heat-stable lantibiotic produced by the tomato pathogen *Clavibacter michiganensis* subsp. *michiganensis*, which inhibits the potato phytopathogen *Clavibacter michiganensis* subsp. *sepedonicus* at nanomolar concentrations. Structurally, this lanthipeptide is composed of two MeLan and one Lan bridge. Compared with actagardine, michiganin A lacks the sulfoxide bond and possesses two substitutions in the residues 5 and 15, a Val and Val in actagardine and Leu and an Ile in michiganin [52–54].

### Cinnamycin-group: ***duramycin, cinnamycin, mathermycin and kyamicin***

Other representative members from class II lanthipeptides are duramycin, cinnamycin, and mathermycin (cinnamycin-type lantibiotic), the first two produced by *Streptomyces*, and the last one identified in the marine actinomycete *Marinactinospora thermotolerans* by genome mining [55, 56]. Another member of this group, kyamicin was identified in a *Saccharopolyspora* strain isolated from ants (*Tetraponera penzigi*) collected in Kenya [57]. These lanthipeptides contain the unusual post-translational modification lysinoalanine, resulting from the cross-linking between Lys19 and Ser6 and a β-hydroxyaspartic acid produced from the hydroxylation of Asp15. Their structure includes 19 amino acids and is formed by four covalent intramolecular bridges. In addition to lysinoalanine, there is one lanthionine and two methyl-lanthionines. Duramycin and cinnamycin share a high degree of sequence and structural homology differing in a single amino acid (Lys2 in duramycin and Arg2 for cinnamycin) [55, 58], while mathermycin exhibits six amino acid substitutions [56, 57].

The mechanism of action of these peptides involves the binding to phosphatidylethanolamine (PE) receptor, a major lipid component of the cellular membrane, showing activity against Gram-positive bacteria [59, 60]. PE is an abundant structural phospholipid present not only in microbial membranes but also in mammalian biological membranes, where it participates in physiological processes such as cell death, cell division, and coagulation [61]. In this line, it has been suggested the use of duramycin and mathermycin as potential candidates for cancer therapy [60, 62]. Mathermycin targets PE in tumor cells in a selective manner compared to normal cells. In normal cells, PE is located only in the inner leaflet of the plasma membrane but in tumor cells, it is found in both, the inner and outer leaflets of the membrane. Besides, mathermycin also shows cytotoxic activity against multidrug-resistant cancer cells, likely by inhibiting mitochondrial function [60].

Duramycin has been found to promote Ca^2+^ release in ovarian and pancreatic cancer cell lines [60, 62]. Indeed, targeted photodynamic therapy with duramycin, can induce cancer cell death and improve the effect of other treatments, including surgery, chemotherapy, or radiotherapy. The selectivity of this approach might increase the coupling of a photosensitizer with tumor-targeted agents such as duramycin to minimize damage to surrounding tissue [63].

The therapeutic activity of duramycin for the treatment of cystic fibrosis has also been investigated. This disease is a recessive genetic condition characterized by loss-of-function mutations in the coding gene for the anion channel cystic fibrosis transmembrane conductance regulator (CFTR), which causes disrupted chloride ion transport and is associated with pulmonary dysfunction that restricts hydration of the airway mucosa. It has been suggested that duramycin promotes chloride secretion over lung epithelial cells, which leads to mucus clearance from the lungs, probably due to unspecific changes in the cell membrane or its components, producing an increase in plasma membrane permeability. This compound positively affects lung function in a phase II study in adolescents and adults with cystic fibrosis under duramycin treatment [64, 65]. A recent study could not determine the beneficial effect of the duramycin treatment compared to a placebo. These conflicting results suggested that the therapeutic range of duramycin is narrow or that the treatment period may need to be longer [66].

Furthermore, duramycin also prevents the entry of viruses such as West Nile, dengue, and Ebola into Jurkat cells. Some enveloped viruses use phosphatidylserine (PS) and PE receptors on host cell membranes to enhance virus entry to the cells [67]. T-cell Ig and mucin domain (TIM) serve as PS receptors promoting phagocytosis of apoptotic cells. Targeting these receptors implies virus access into the host cell. Therefore, the inhibition of infection by the virus attachment to the receptor TIM1 is likely duramycin’s primary mechanism of action as an antiviral agent [68].

The application range of duramycin and cinnamycin is beyond their use as therapeutic peptides. For instance, these two lanthipeptides have been used to analyze the distribution and location of PE in target cells and tissues. This is because duramycin and cinnamycin contain two and one primary amines in the N-terminal region, respectively, enabling covalent reactions without interfering with the PE binding site. Their binding capacity has also been exploited for their use as molecular probes for the detection of this phospholipid due to its low molecular weight (2,013 Da and 2,041 Da, respectively), high binding affinity, high specificity, and stable structure to thermal and proteolytic degradation [69]. Duramycin has been assessed as a molecular probe candidate for in vivo imaging applications radiolabelled with Technitium-99 m (99mTc). This probe exhibits favorable clearance profiles and tissue penetration [70]. 99mTc-duramycin imaging is a promising approach for assessing the early tumor response for anticancer treatment. In apoptotic cells, PE is externalized on the cell surface, whereby the detection of this compound can be used as a molecular marker. The use of novel non-invasive tools for tissue imaging is of great importance for predicting tumor response to treatment, designing individualized and optimized therapies, and avoiding unnecessary side effects caused by exposure to aggressive treatments [71].

### Novel two-component lantibiotics: ***roseocin and birimositide***

Roseocin is a two-component lantibiotic identified by genome mining of *Streptomyces roseosporus* NRRL 11,379. This lantibiotic harbors an α-peptide with four methyl-lanthionine rings and a disulfide bond necessary for the antimicrobial activity. In addition, a β-peptide composed of six methyl-lanthionine rings is required with post-translational modifications carried out by a single lanthionine synthetase (RosM). The presence of both peptides is necessary to observe a synergistic antimicrobial activity against the resistant Gram-positive bacteria pathogens [53]. Lately, through a phylogeny-based genome mining study, four Rosα variants were obtained where the Leu-8-Phe substitution in Rosα showed four-fold lower MIC against methicillin-sensitive *Staphylococcus aureus* (MSSA) ATCC 25,923 (combined with Rosβ), which demonstrates that developing variants of one of the components of this class of lanthipeptides, can generate powerful products [72].

Simultaneously, Walker and co-workers identified a two-component lantipeptide termed birimositide formed by the Brtα and Brtβ peptides and produced by *Streptomyces rimosus* subsp. *rimosus* WC3908. Like two-component lanthipeptides isolated in lactic acid bacteria (LAB), α- and β-peptides exhibit a Ser to D-Ala conversion, presumably performed by a luciferase-like monooxygenase present in the BGC. As with roseocin, synergistic antibacterial activity against Gram-positive bacteria was reported [73, 74].

## Class III lanthipeptides

### Labyrinthopeptins

Labyrinthopeptins are members of class III lanthipeptides isolated by Aventis Pharma from the desert bacteria *Actinomadura namibiensis* DSM 6313 [75]. The main structural feature of this kind of lanthipeptides is the carbacyclic post-translationally modified tri-amino acid labionin (Lab) and a disulfide bond. These compounds are ribosomally synthesized as prepropeptides from the genes labA1 (LabA1 and LabA3) and labA2 and then post-translationally modified by the tri-domain enzyme LanKC [76]. The final steps in biosynthesis are the cleavage of the leader peptide, the disulfide bond formation, and the export into the extracellular space. The labyrinthopeptin A2 (LabA2) has a potential application in the treatment of neuropathic pain due to its activity observed in a spared nerve injury mouse model [77], while the labyrinthopeptin A1 (LabA1) displays antiviral activity against HSV-1 and HIV-1. Remarkably, when it is combined with clinically approved antiretroviral drugs, a synergistic activity is observed. Concerning the anti-HIV mechanism of action, LabA1 is a viral entry inhibitor that interacts with the protein gp120 in the viral envelope. Furthermore, it inhibits cell-to-cell transmission, which may have application in preventing sexually transmitted diseases. Likewise, this compound preserves its activity even against drug-resistant HIV strains and does not have a negative effect on vaginal Lactobacilli populations or on the endometrial and cervical epithelial cells, which further supports its potential use [78].

In addition, LabA1 and LabA2 have been shown to possess antiviral activity against a broad range of enveloped viruses, including respiratory syncytial virus (RSV), dengue virus, Zika virus, West Nile virus, hepatitis C virus, chikungunya virus, Kaposi’s sarcoma-associated herpesvirus, cytomegalovirus, and herpes simplex virus. LabA1 and LabA2 exhibit a synergistic antiviral effect and are effective in low concentrations, making them promising candidates for prophylactic or therapeutic treatment of viral infections. Their mode of action involves binding to phosphatidylethanolamine in the virus membrane, which disrupts its integrity without being affected by resistance mutations to other RSV inhibitors. Additionally, these compounds demonstrate stability, favorable pharmacokinetic properties, and low cytotoxicity in murine models, and have been shown to be an effective antiviral agent [79].

### NAI-112

NAI-112 is a glycosylated lanthipeptide produced by *Actinoplanes* DSM24059, comprised of 22 amino acids and neutrally charged. Its unique structure includes new modifications not observed before in lanthipeptides, such as a 6-deoxyhexose moiety N-linked to a tryptophan residue and a C-terminal methyl-labionin (MeLab) in addition to an N-terminal Lab. Likewise, NAI-112 has shown its therapeutic potential by reducing pain symptoms in mice nociceptive pain models [80].

It is worth mentioning that antibacterial activity has also been described in class III, such as the case of avermipeptin B. This 24-amino acid peptide was detected by genomic mining in *Streptomyces actuosus* ATCC 25,421 and produced by heterologous expression in *Streptomyces lividans* TK24. Avermipeptin B is an analog of avermipeptin produced in *Streptomyces avermitilis* DSM 46,492. In vitro, assays demonstrated antibacterial activity against gram-positive bacteria like *Staphylococcus aureus* [81, 82]. Likewise, the lantipeptide NAI-112 showed weak antibacterial activity in vitro against staphylococci and streptococci bacteria.

### Class IV lanthipeptides

#### Venezuelin-like lanthipeptides

Class IV is the least studied and characterized group of lanthipeptides, having different biological properties to antimicrobial activity [24]. Unlike other lanthipeptides, the identification of these compounds has been solely by bioinformatic analysis, allowing only their heterologous production since isolation from the natural producer has not been possible [83, 84]. A few members of this class have been identified, being venezuelin the first and most representative class IV lanthipeptide. This peptide is not produced under standard culture conditions despite detecting the venezuelin biosynthetic cluster in *Streptomyces venezuelae*. To determine the biological activity of this lanthipeptide, the biosynthetic enzymes were produced in vitro along with engineered VenA (LanA) mutants with protease recognition sites. However, none of the produced variants showed antimicrobial activity [84, 85]. Albeit new venezuelin-like lanthipeptides have been identified, only the streptocollin produced by *Streptomyces collinus Tü 365* shows biological activity, since it seems to act as a moderate inhibitor of the protein tyrosine phosphatase 1B (PTP1B), which it is involved in insulin and leptin signaling. Therefore, streptocollin is being considered as a therapeutic agent to treat obesity and diabetes [20, 84, 87, 88].

### Class V lanthipeptides

#### Cacaoidin, lexapeptide and pristinin A3

Class V members have a unique combination of inherent features from lanthipeptides and linaridins. Furthermore, the dehydroamino acids and the lanthionine ring formation seem to be catalyzed by three monofunctional proteins without homology with the synthetases previously identified in other classes [3]. The LanK and LanY dehydratases catalyze the dehydration of Ser/Thr. The conjugate addition of a Cys residue onto the dehydro amino acids (Dha/Dhb) to form the AviCys and (Me)Lan rings is performed by a flavin-dependent decarboxylase (LanD). The final cyclization step to construct final products energetically favored seems to occur spontaneously. However, in a recent report, a lanthipeptide cyclase (LanKXY) is necessary to form an energetically favored final product [21]. These authors used bioinformatic tools to identify more than 240 putative class V lanthipeptide clusters with a LanC cyclase. By reconstitution studies, they demonstrated that the final cyclase-catalyzed product was clearly distinct from that formed spontaneously.

Regarding linaridins, they are an understudied class of RiPS, with only a few members described as exhibiting antimicrobial and antitumor activity [88]. They are linear, dehydrated peptides characterized by the presence of dehydrobutirine, a dehydrated alkene-containing amino acid derived from threonine, catalyzed by a currently unknown enzyme, and an AviCys catalyzed by linaridine decarboxylase LinD. Some class V lanthibiotics, also exhibit Nα,Nα-dimethylation of the N-terminus, by a methyltransferase (LinM) activity [89].

Cacaoidin, lexapeptide and pristinin A3 are three representative members of this class identified from *Streptomyces cacaoi* CA-170,360, *Streptomyces rochei Sal35*, and *Streptomyces pristinaespiralis* ATCC 25,468, respectively. Cacaoidin possesses activity against Gram-positive pathogens, including MRSA and *Clostridium difficile* [90, 91], whereas lexapitide displays a broader activity spectrum against methicillin-resistant *S. epidermidis* (MRSE), *Enterococcus faecalis*, *M. smegmatis* mc2155, just as with MRSA; so far, no bioactivity of pristinin A3 has been reported [92]. Likewise, all members of this group have notable posttranslational modifications such as a C-terminal AviMeCys and N-terminal N,N-dimethylation. Cacaoidin contains a glycosylated tyrosine residue, D-aminobutyric acid, and D-Ala residues, as with the antibacterial Lexapeptide. Recent in vitro studies have shown that in addition to binding lipid II, cacaoidin may also act by inhibiting the murein transglycosylase domain of the penicillin-binding protein (PBP2) of *S. aureus*, suggesting a synergistic antibacterial effect for cacaoidin joining two distinct targets [93].

### Concluding remarks and future challenges

The main health-related challenges for the 21st century are the emerging crisis of antibiotic resistance and the increasing cancer incidence, which must be faced by discovering novel drugs. Natural products (NPs) from microorganisms represent a valuable source of such drugs, many of which are currently on the market as antibiotics and anti-cancer agents [94]. Unfortunately, natural product-based drug discovery efforts declined in recent years due to frequent re-discovery of already-known compounds and high costs in screening and drug development [95]. In this sense, *Actinomycetes* represent an invaluable source of these compounds that have not yet been explored in detail. Around 80% of known actinobacterial compounds are produced by this group of microbes, of which approximately 10,000 show antibacterial activity [16].

To date, only a small fraction of known active compounds have been related to the biosynthetic gene clusters (BGCs) encoding enzymes for the biosynthesis of these NPs. Bioinformatics analyses based on genome sequencing data from *Actinomycetes* indicate the enormous diversity of BGCs encoded for lanthipeptides of different classes. A study by Belknap and co-workers analyzed 1,110 streptomycetes genomes and found the presence of these clusters in 540 species, which places lanthipeptides within the five BGCs with the highest abundance in this bacterial group and the largest group of RiPPs [96]. In another study focused on searching RiPPs within 629 actinobacterial genomes, it was found that class III lanthipeptides represent the class with the highest abundance, followed by classes I, II, and IV [14], showing that these peptides are widely distributed in *Actinobacteria* and that many BGCs are silent under laboratory conditions, which has hampered the discovery of new molecules. Many of these BGCs encode lanthipeptides, which harbor many structures and functions. These lanthipeptides have been studied mainly as antimicrobials.

The lack of standardized and well-characterized genetic parts, as well as synthetic biology tools to engineer either actinomycetes (natural producers) or the key players in the biosynthesis of lanthipeptides, have been the major bottlenecks for the discovery of novel natural products with different therapeutic activities. However, this gap has been closed in recent years due to dropping costs in sequencing and DNA synthesis coupled with synthetic biology technologies. These conditions have fostered the development of new strategies to produce and discover new compounds relevant to the therapeutic area. Regarding these technologies in the lanthipeptide field, in 2020, Ran Liu and co-workers [97] developed a rapid and high-throughput screening strategy based on cell-free extracts (in vitro transcription and translation technology) from *E. coli* to identify novel and functional antimicrobial lanthipeptides. Using this technology, they reported a nisin mutant with stronger activity against Gram-negative bacteria and a nisin analog with more intense antimicrobial activity than nisin itself [97]. This revolutionary technology has also produced other mature RiPPs such as lactazole, goadsporinm, thiocillin, and lasso peptides. From these latter, Si et al. [98], demonstrated the capability to produce several sequence-diverse lasso peptides using cell-free technology, having as an entry point the lasso-forming cyclase from the fusilassin pathway.

Recently, the lanthipeptide engineering based on the use of post-translational modifier biosynthetic enzymes for adding functional groups and moieties, such as halogenation and methylation on the amino acid chain, has allowed the expansion of the structural diversity and biological activities of the lanthipeptides. Hence, this strategy is a promising biocatalytic tool since post-translational modifications provide the biological features of lanthipeptides by regulating the affinity to specific biological targets. Accordingly, these modifications are relevant for engineering lanthipeptides with therapeutic applications [99].

Scientists from academia and industry have promoted clinical evaluation of some actinobacterial lanthipeptides like actagardine, duramycin, microbisporicin, and mutacin 1140, detecting attractive pharmacokinetic profiles in these compounds [40]. In preclinical studies, NVB333, tested for treating bacterial infections, has shown good pharmacological and pharmacokinetic properties [17, 50].

Regarding marketing, several commercially available lanthipeptides include nisin, subtilin, gallidermin, planosporicin, NAI-107 (microbisporicin), actagardin, duramycin, and others. Most of them are for livestock applications as antimicrobials, although some also exhibit immunomodulatory, anticancer, antiallodynic, and antinociceptive activities [6]. Contrary to nisin and subtilin, which several companies produce on a big scale for food industry applications at a competitive cost, most are made on a trim level and are available at a high price.

Therefore, future research should be conducted to systemize high-throughput strategies for screening potential candidates considering vanguard technologies from different disciplines around biology engineering [4, 100, 101], including the exploitation of lanthipeptides biosynthetic enzymes modularity for in vivo engineering and generation of new diverse structures and activities [99]. To expand potential applications of lanthipeptides, like, for example, the newly discovered anti-inflammatory effect of myxococin [102], the pinenins, first antifungal lantibiotics [103] or the archalan-α active against halophilic archaea [104]. It is also required to focus on the action mode of these molecules to improve their activities. To synchronize the expression systems and fermentation conditions for scaling-up process to produce them at feasible yields to enter the commercial market.

Lanthipeptides are stunning structures that, besides their ability to kill bacteria and fungi, exhibit additional activities of potential application in the medical field. Generally, they are stable to extreme temperatures and pH values, making these compounds attractive to the pharmaceutical industry. Therefore, they merit to be continuously studied. Fortunately, many scientific groups worldwide are working to contribute solutions to the challenges mentioned above. Therefore, a bright and promising future is likely for these small and amazing compounds.

## Data Availability

Not applicable.
